# Endoscopy simulation for pre-clerkship students

**DOI:** 10.36834/cmej.67898

**Published:** 2020-03-16

**Authors:** Amit R. Persad, Lalit K. Verma, Rabindranath Persad

**Affiliations:** 1 Division of Neurosurgery, University of Saskatchewan, Saskatchewan, Canada; 2 Division of Pediatric Gastroenterology, University of Alberta, Alberta, Canada

## Abstract

Here we report a simulation session carried out with pre-clerkship medical students during their gastroenterology block. We used endoscopy simulator to cement the clinical and anatomic implications of endoscopy and to build interest in gastroenterology. Students thought the session was helpful for their interest and understanding. Endoscopy simulation provided for pre-clinical students is an enjoyable adjunct to gastroenterology learning.

## Déclaration des répercussions

Nous rapportons ici une séance de simulation réaliséeavec des étudiants en médecine au pré-externatet pendant leur bloc de gastro-entérologie. Nous utilisons un simulateur d’endoscopie pour consoliderles implications cliniques et anatomiques de l’endoscopie et pour susciter un intérêt pour la gastro-entérologie. Les étudiants ont considéré la séance comme utile pour leurs intérêts et leur compréhension. Une simulation d’endoscopie offerte aux étudiants fu pré-externat est un complément agréable à leur apprentissage en gastro-entérologie.

## Background

Medical simulations help trainees develop practical knowledge and skills.^1^^,^^2^ A previous study demonstrated that endoscopic simulation is useful for third year clerks during their gastroenterology rotation.^2^ Endoscopy simulation can also be used for testing^3^ and training visuospatial tasks. We found no studies assessing virtual reality simulators with pre-clinical students. We ran an endoscopy simulation session with pre-clinical students in order to assess student satisfaction.

## Methods

We conducted a prospective observational study using qualitative methodology to evaluate an endoscopy simulation session for pre-clinical medical students. We sent invitations to the Oncology Club at the University of Alberta, consisting of 142 pre-clerkship medical students.

We used the CAE Healthcare EndoVR simulator. It consists of a computer module with openings simulating the oral and anal orifices, a screen displaying the VR environment, and a console. We used cases of peptic ulcer disease and chemotherapy-related esophagitis.

We held a one-hour session facilitated by a pediatric gastroenterologist and gastrointestinal (GI) fellow. After a 15-minute orientation, students performed a supervised simulation with the goal of assessing endoscopic anatomy, reaching the duodenum, and providing a diagnosis. Post-simulation, the authors discussed the case and potential complications with the participants. Students completed a 5-point Likert scale (1-strongly disagree, 2-disagree, 3-neutral, 4-agree, 5-strongly agree) evaluation and provided comments. Data are reported as medians with interquartile ranges.

## Results

We invited 142 pre-clinical medical students, 86 in second-year and 56 in first-year. Twenty-five students initially responded (17.6%), twenty-two in second-year and three in first-year. We confirmed attendance one week before the session. Nine second-year students (40.9% of respondents) and one first-year student (33% of respondents) confirmed attendance (n=10).

Evaluations are summarized in Table 1. Cronbach’s alpha was calculated using SPSS (α=0.969), and indicated high internal validity. Written comments indicated that students found the session enjoyable and useful. Students requested GI bleed and diarrhea simulations in the future.

**Table 1:** Feedback to session (n=10)

	Median	Interquartile Range (Q1, Q3)
I enjoyed this session	5	5, 5
The session was the right length of time.	5	5, 5
The group size was appropriate.	5	5, 5
This session built on my understanding of interventional gastroenterology	5	4.25, 5
I was able to correlate my personal knowledge to findings during the session	5	4, 5
This session increased my interest in gastroenterology	4.5	3.25, 5
This session improved my understanding of endoscopy as a diagnostic tool	5	5, 5
This session improved my understanding of endoscopy as a therapeutic tool	5	4.25, 5
This session allowed me to understand the use of an endoscope	5	5, 5
My knowledge of complications of endoscopy and their management was enhanced.	5	5, 5
My understanding of GI anatomy was enhanced by this session	5	5, 5
My understanding of GI pathology was enhanced by this session	5	5, 5
I would have valued this type of session during the GI block	4.5	4, 5
I would be interested in having access to this type of simulation system during GI rotations in clerkship	5	4, 5
The instructor provided clear instructions for success at the station	5	5, 5
The instructor explained usage of the instrument as well as findings during the simulation	5	5, 5
The instructor was familiar with the equipment and solved technical issues promptly	5	5, 5
Overall, the session was excellent	5	5, 5

## Discussion

Medical education employs various simulation-based techniques including virtual patient softwares^4^^,^^5^ and high-fidelity simulations.^1^^-^^3^ Endoscopy simulation is typically reserved for residents and fellows.^3^ Previous work documents experience with medical students in their clerkship years.^2^

While medical students will not have the opportunity to translate endoscopic skills until a later stage of training, hands-on learning and understanding of the process of endoscopy proved to be a valuable experience. Teaching anatomy in an applied manner helps trainees contextualize basic science knowledge^6^

At the University of Alberta, gastroenterology is a pre-clinical block with endoscopy shadowing available. Students shadow endoscopy to visually understand gastroenterology. Students at this session commented that it was a good adjunct to shadowing.

Future sessions may integrate interactive patient simulation into the session as suggested previously^4^. Advances in simulation of clinical experience using natural language interaction may enhance this.^5^

We were limited by the small sample size, qualitative data, and sampling bias as the session was voluntary.

### Conclusion

Our small pre-clinical student cohort found endoscopy simulation to be an enjoyable and useful adjunct to their gastroenterology block.
